# A Systematic Literature Review of Substance-Use Prevention Programs Amongst Refugee Youth

**DOI:** 10.1007/s10597-024-01267-6

**Published:** 2024-04-09

**Authors:** Elijah Aleer, Khorshed Alam, Afzalur Rashid

**Affiliations:** 1 Faculty of Business, Education, Law and Arts, School of Business, University of Southern Queensland, Toowoomba, QLD 4350 Australia; 2 Faculty of Business, Education, Law and Arts, School of Business, Centre for Health Research, University of Southern Queensland, Toowoomba, QLD 4350 Australia

**Keywords:** Refugee youth, Systematic literature review, Substance use prevention programs

## Abstract

This paper aims at exploring existing literature on substance use prevention programs, focusing on refugee youth. A comprehensive search for relevant articles was conducted on Scopus, PubMed, and EBSCOhost Megafile databases including Academic Search Ultimate, APA PsycArticles, APA PsycInfo, CINAHL with Full Text, E-Journals, Humanities Source Ultimate, Psychology and Behavioural Sciences Collection, and Sociology Source Ultimate. Initially, a total of 485 studies were retrieved; nine papers were retained for quality assessment after removing duplicates. Of the nine studies that met the inclusion criteria, only three are found to partially addressed substance use prevention programs. The two substance use prevention programs that emerge from the study are Adelante Social and Marketing Campaign (ASMC), and Screening and Brief Intervention (SBI). Six others explored protective factors and strategies for preventing substance use. The study findings show that refugee youth held negative attitudes toward institutions that provide substance use prevention programs. This review concluded that refugee youth often experience persistent substance use as they are not aware of prevention programs that may reduce the prevalence and/or severity of such misuse.

## Introduction

Increasingly, literature suggests that refugee youth face a heightened vulnerability to substance use, coupled with a limited awareness of substance use prevention programs. Refugees’ susceptibility to substance use is linked to adverse living conditions and maladaptive coping mechanisms (Posselt et al., 2015; Ramachandran et al., 2019; Roberts et al., 2011). As a result, research suggests that the prevalence of substance use amongst refugees ranges from 17 to 37% in camps and 4% to7% in the community setting (Horyniak et al., 2016a). Another study revealed that 14.9% of men and 0.7% of women from refugee background exhibited substance use (Ramachandran et al., 2019). The concerning aspect of this situation lies in the fact that substance use and its associated risks are well-documented within refugee setting (Gire et al., 2019; Luitel et al., 2013), with a growing call to integrate substance use prevention programs into refugee services due to the prevalence of the phenomenon (Horyniak et al., 2016a). Such recommendation emphasises the importance of addressing the knowledge gap on substance use prevention programs amongst the refugee youth. Research indicates that if the substance use prevention programs are not made known to those at risk individuals, it could have detrimental effects on such individuals (Bauman and Phongsavan, 1999). Failure to address the knowledge gap of substance use prevention programs could place, refugee youth at an increasing risk of various negative outcomes such as disorder, higher mortality, accidental injury, liver diseases, violence, dysfunctional work, and school dropout due to substance use (Ji et al., 2021; Kuntsche et al., 2017; Li et al., 2017; Momeñe et al., 2021). Hence, it is important to document the knowledge of substance use prevention programs amongst refugee youth in the literature to ensure that the groups are informed about the negative consequences.

As per this study, substance use prevention programs refer to a myriad of substance-free and medication treatments administered to assist individuals to reduce substance use (Alayan et al., 2021). While substance use refers to as a prolonged harmful use of any substance, which can result in problems such as non-fulfilling social roles, withdrawal and tolerance symptoms, substance use disorders and attributable to burden of disease and mortality (American Psychiatric Association, 2013; Rehm et al., 2013). In this case, substances can include alcohol, cannabis, methamphetamine and other stimulants drugs, non-medical use of pharmaceutical drugs, illicit opioids including heroin, tobacco and other emerging psychoactive substances (AIHW, 2020). In Australia, youth refers to a person aged between 12 and 24 years (AIHW, 2021). Accordingly, refugee youth in this study are those between the ages of 12 and 24.

### Substance Use Prevention Programs

There are several substance use prevention programs in the literature, the aim of which are to reduce harms of substance use. The last two decades have witnessed a surge in studies conducted on substance use prevention programs for different socio-demographic groups that produced information about the initiation, prevalence and associated behavioural, social, and educational outcomes (Fishbein et al., 2006; Gau et al., 2012; Gruenewald et al., 2009; Springer et al., 2004). The surge in research reaffirms that substance use prevention programs play an important role in reducing the consequences of substance use. Notably, there are several factors which permit individuals to engage in use substance. These include peer pressure, poor neighbourhood, inability to cope with difficulties, cultural norms, family history of drug use and lower level of education. Family structure and mental disorder play a vital role in initiation and maintenance of substance use (Gattamorta et al., 2017; Peloso et al., 2021). The knowledge of various factors, that induce individuals to use substances is vital as they play a significant role when designing substance use prevention programs.

Some of the known substance use prevention programs include individual and group counselling, alternative programs, and family and community interventions (Barrett et al., 1988; Foss-Kelly et al., 2021; Radoi, 2014). These programs are designed to influence social and psychological factors associated with the initiation and maintenance of substance use (Barrett et al., 1988). The social factors include peer pressure, a deviation from conventional values. Including those of one’s family, school, and religion, while the psychological characteristics include low self-esteem and an attitude of tolerance towards deviancy (Barrett et al., 1988; Hater et al., 1984; Radoi, 2014). Substance use prevention programs aim to approach social and psychological factors in a unique way depending on their goal and outcome. Each of the factors requires a different approach when designing a substance use prevention program. For example, the primary objective of providing counselling to young individuals who engage in substance use is to assist them in overcoming their low self-esteem and embracing the positive societal norms that are linked to such behaviour (Barrett et al., 1988). The effectiveness of an individual program depends on the participants’ attitude toward intervention and their outcomes (Espada et al., 2015). For instance, participants sometimes refuse to join the prevention program due to fear of being reported to authorities (Kvillemo et al., 2021).

Peer pressure is widely acknowledged as a significant source of the initiation and maintenance of substance use amongst youth. According to social learning theory, youth substance use is a consequence of peer pressures originating from their reference groups (Watkins, 2016). To address the substance use where such pressure is deemed to be the initiation and maintenance factor, group counselling is believed to be a key prevention program (Barrett et al., 1988). This is because peer relations play a powerful influence, and therefore, researchers often use group counselling rather than individual counselling to promote healthy and acceptable relationships, foster social skills, and thus to develop healthy forms of recreational activities amongst peers.

Apart from counselling, adopting alternative programs such as substance-free strategies reduce the initiation and maintenance factors of substance use. Behavioural economic theory suggests that an increase in rewarding substance-free activities can lead to a reduction in substance use (Murphy et al., 2019). The structured substance-free activities approach is based on the relationship between the reinforcement derived from substance-related activities to the reinforcement derived from substance-free activities (Correia et al., 2005). Research shows that substance use programs that are supplemented with either relaxation training or a behavioural economic session focused on increasing substance-free activities are associated with reductions in substance use (Murphy et al., 2019). Notably, increasing substance-free activities is suggested to be useful in substance use prevention in vulnerable youth (Andrabi et al., 2017).

Community, family, academic engagements, work, and religious activities play a significant role in reducing the initiation and maintenance of substance use and its related consequences. Similarly, individual and group counselling, alternative programs, and family and community interventions have also led to a reduction in the initiation and maintenance of substance use amongst youth. Research demonstrated a negative relationship between commitment to conventional values such as family, religion, and education, and substance use amongst the youth (Sussman et al., 2006). This evidence is supported by social bond theory, which postulates that commitment to conventional values of one’s family, religion, and school act to prevent deviant responses (Nijdam-Jones et al., 2015). Similarly, the Family Interaction Theory suggests that social learning, parent attachment, and intrapersonal characteristics equally discourage youth risk-taking behaviours (Ismayilova et al., 2019). The evidence appeared in several substance use prevention programs (Huang et al., 2014; Ishaak et al., 2015; Liddle et al., 2006). For instance, the Adolescent Day Treatment Program (ADTP) in Canada implements a social learning approach stressing positive support for appropriate substance, anti-social coping behaviour, and social skills (Liddle et al., 2006).

Some substance use prevention programs are designed to assist individuals with the development of skills and attitudes through a community approach. The approach has seen youth cessation of substance use and helped them make changes leading to substance-free lifestyles (Wade-Mdivanian et al., 2016). One of the substance use prevention programs, which adopts a community approach is Multidimensional Family Treatment (DFT). DFT targets the initiation and maintenance of youth substance use by addressing coping strategies, parenting practices, other family members, and interactional patterns that contribute to the continuation of substance use and related consequences (Liddle et al., 2006). DFT also addresses the functioning of youth and family using the social systems influencing the youth’s life such as school, work, peer networks, and the juvenile justice system (Liddle et al., 2006; Valente et al., 2007). In support of the community approach, researchers argue for the inclusion of the perspectives of community members in substance use prevention programs because they understand the unique needs of the people with whom they share a bond (Bermea et al., 2019). Researchers also focus the interconnected nature of their socio-environmental relationships that can facilitate advocacy for change at the community level (Bermea et al., 2019).

### Research Gap

Despite the vast knowledge of substance use prevention programs in the literature, research on the refugee youth remains scarce. The lack of research on substance use prevention programs for refugee youth may be due to many factors. First, scholars might have ignored the severity of the issues amongst the groups. Secondly, the socio-economic benefits of the prevention programs might have been underestimated in the literature. Thirdly, the political aspect of substance use prevention programs for refugee youth might have not been thoroughly evaluated in the policy frameworks. The socio-economic benefit of substance use prevention programs underscores a pressing need to begin synthesizing evidence given the deleterious nature of substance use if it is left unmitigated. The knowledge of substance use prevention programs is significant to vulnerable groups like refugee youth because they seek assistance whenever they succumb to substance use. As a result, they will avoid the negative consequences of substance use and subsequently exploit the social benefit. Furthermore, the knowledge of substance use prevention programs can assist organisations and advocacy groups assisting refugee youth to provide them with better services.

This study aims at contributing to substance use prevention programs literature by conducting a systematic literature review to synthesize evidence on such programs, their attitudes towards the program, and amongst refugee youth to fill the gaps in knowledge and provide directions for future research.

### Research Questions

The following questions are designed to achieve the aims and objectives of the systematic literature review:

#### RQ1

What different substance use prevention programs are used to assist refugee youth with substance use?

#### RQ2

What is the refugee youth’s attitude toward substance use prevention programs?

#### RQ3

What are the outcomes of a substance use prevention program?

## Method

To ensure the validity and reliability of this study, systematic review guidelines are followed (Toews, 2017). This is because the systematic review is useful in mapping out areas of uncertainty, identifying the lack of research on a particular topic, and pointing out an area where research is needed (Rethlefsen et al., 2021). The systematic review method provides complete and accurate reporting, which facilitates assessment of how well reviews have been conducted (Toews, 2017).

Unlike a traditional review, a systematic review uses a transparent, replicable, and scientific steps purposely to mitigate the risk of bias by conducting a comprehensive literature search and providing an audit trail of procedures, decisions, and conclusions (Caldwell and Bennett, 2020). The systematic review reports a reproducible search strategy that increases the reliability and validity of the study.

By following systematic review guidelines, this study will mitigate bias and increase its validity and reliability. The following steps are adopted to conduct the systematic review:

### Step 1: Identifying Keywords

To synthesize the evidence of substance use prevention programs available in the literature amongst refugee youth, a database search began with a simple string of “substance use AND Prevention AND Refugee AND youth” in the library. Then other search terms were obtained using a permutation of the keywords in EBSCOhost Megafile Ultimate (Table 1).

**Table 1:** Keywords and search strings

Concept	Keywords and strings	Boolean operators
Substance Abuse	(“substance abuse” OR “drug abuse” OR “alcohol abuse” OR addiction OR narcotics OR “substance use” OR “illicit drug” OR “drug use” )	AND
Prevention program	(prevention OR intervention OR treatment OR program OR therapy)	AND
Refugee	(refugees OR “asylum seekers” OR “displaced people”)	AND
Youth	(youth OR adolescent OR “young adult” OR teen* OR “young people”)	AND

### Step 2: Search Strategy

In the next step, a comprehensive search for relevant articles was conducted on 12th of October 2021 on three major databases: Scopus, PubMed, and EBSCOhost Megafile databases including Academic Search Ultimate, APA PsycArticles, APA PsycInfo, CINAHL with Full Text, E-Journals, Humanities Source Ultimate, Psychology and Behavioural Sciences Collection, and Sociology Source Ultimate. A total of 485 studies were retrieved following the comprehensive search of the databases (Table 2).

**Table 2:** Search log

Date	Search strategy	Database	Number of results	Field search	Limits	Notes	Alert? Y/N
9/17/2021	(“Substance abuse” OR “drug abuse” OR “alcohol abuse”) AND (“prevention program” OR intervention) AND refugee* AND (youth OR teenager)	Library search	5686	All	Nil		
9/17/2021	(“Substance abuse” OR “drug abuse” OR “alcohol abuse”) AND (“prevention program” OR intervention) AND refugee* AND (youth OR teenager)	Psycinfo	6	All	Nil		
22/09/21	( “substance abuse” OR “drug abuse” OR “alcohol abuse” OR addiction OR narcotics OR “substance use” OR “illicit drug” OR “drug use” ) AND ( prevention OR intervention OR treatment OR program OR therapy ) AND ( refugees OR “asylum seekers” OR “displaced people” ) AND ( youth OR adolescent OR “young adult” OR teen* OR “young people” )	Academic Search Ultimate, APA PsycArticles, APA PsycInfo, Psychology and Behavioral Sciences Collection, Sociology Source Ultimate	93	all			
22/09/22	( “substance abuse” OR “drug abuse” OR “alcohol abuse” OR addiction OR narcotics OR “substance use” OR “illicit drug” OR “drug use” ) AND ( prevention OR intervention OR treatment OR program OR therapy ) AND ( refugees OR “asylum seekers” OR “displaced people” ) AND ( youth OR adolescent OR “young adult” OR teen* OR “young people” )	Academic Search Ultimate, APA PsycArticles, APA PsycInfo, Psychology and Behavioral Sciences Collection, Sociology Source Ultimate	78	all	peer-reviewed		
22/09/21	( “substance abuse” OR “drug abuse” OR “alcohol abuse” OR addiction OR narcotics OR “substance use” OR “illicit drug” OR “drug use” ) AND ( prevention OR intervention OR treatment OR program OR therapy ) AND ( refugees OR “asylum seekers” OR “displaced people” ) AND ( youth OR adolescent OR “young adult” OR teen* OR “young people” )	Scopus	90	All			
22/09/21	(((prevention OR intervention OR treatment OR program OR therapy) AND (Adolescent [MeSH Terms])) AND (Refugees[MeSH Terms])) AND (Substance-Related Disorders[MeSH Terms])	PubMed	27				
12/10/2021	( “substance abuse” OR “drug abuse” OR “alcohol abuse” OR addiction OR narcotics OR “substance use” OR “illicit drug” OR “drug use” ) AND ( prevention OR intervention OR treatment OR program OR therapy ) AND ( refugees OR “asylum seekers” OR “displaced people” ) AND ( youth OR adolescent OR “young adult” OR teen* OR “young people” )	Academic Search Ultimate, APA PsycArticles, APA PsycInfo, Psychology and Behavioral Sciences Collection, Sociology Source Ultimate	291	All	peer reviewed, English		
12/10/2021	( “substance abuse” OR “drug abuse” OR “alcohol abuse” OR addiction OR narcotics OR “substance use” OR “illicit drug” OR “drug use” ) AND ( prevention OR intervention OR treatment OR program OR therapy ) AND ( refugees OR “asylum seekers” OR “displaced people” ) AND ( youth OR adolescent OR “young adult” OR teen* OR “young people” )	PubMed	111	All	Peer reviewed, English		
12/10/2021	( “substance abuse” OR “drug abuse” OR “alcohol abuse” OR addiction OR narcotics OR “substance use” OR “illicit drug” OR “drug use” ) AND ( prevention OR intervention OR treatment OR program OR therapy ) AND ( refugees OR “asylum seekers” OR “displaced people” ) AND ( youth OR adolescent OR “young adult” OR teen* OR “young people” )	Scopus	83	All	Peer reviewed, English		

### Study Selection

**Fig. 1:**
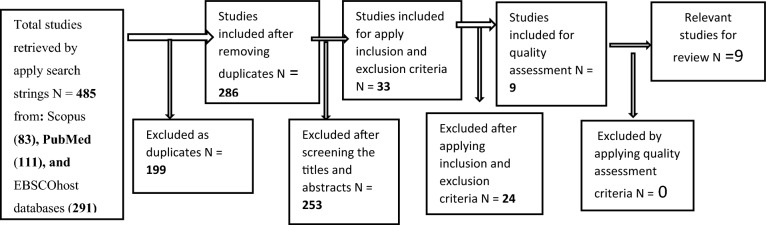
PRISMA of workflow

All the retrieved studies were exported to Endnote X9, and 199 duplicates were removed. The titles and abstracts of the remaining 286 studies were reviewed and 253 studies were excluded for not focusing on substance use prevention programs. A total of 33 studies were further screened using inclusion and exclusion criteria. As a result of the exercise, 24 studies were excluded and nine were included for quality assessment. The PRISMA workflow diagram below shows the process of identifying and selecting eligible studies for this systematic review (Fig. 1). The data visualisation displays identified, included, and excluded papers and their explanations.

### Exclusion and Inclusion Criteria

This systematic literature review on substance use prevention programs amongst refugee youth was conducted after adopting exclusion and inclusion criteria. To assist in the process of selecting relevant studies in this systematic literature review, studies were limited to peer-reviewed articles published in the English language. Unpublished articles were excluded, and no restriction was placed on the date of publication of the studies.

Furthermore, the selection of articles was restricted to the following eligibility criteria:

### Inclusion Criteria

- Studies that explored substance use and prevention/reduction/treatment/intervention programs amongst refugee youth.
- Studies that explored substance use amongst refugee youth included another perspective of substance use prevention programs.
- Studies that investigated and reported motivation for substance use refugee youth.

### Exclusion Criteria

- Studies that addressed substance use but did not include any intervention.
- Studies that addressed substance use prevention and never mentioned refugee youth.
- Studies that addressed substance use prevention programs amongst refugees in general.
- Studies that addressed immigrant youth but did not mention refugees.

### Quality Assessment

The quality of studies included in the systematic literature review was evaluated using the Strengthening the Reporting of Observational Studies in Epidemiology (STROBE) statement checklist (von Elm et al., 2007). This quality assessment tool is chosen for this study because to its usefulness and applicability to all studies (Vandenbroucke et al., 2014; von Elm et al., 2007). The explanation and elaboration of the different components of the STROBE provide readers with a clear understanding of the study (Vandenbroucke et al., 2014).

A total of twenty STROBE items from the checklist were used to assess the quality of the studies. These include 1 A. title, 1B. abstract, 2. background/rationale, 3. objective, 4. design, 5. setting, 6. eligibility of the participants, 7. variables, 8. data source/measurement, 10. study size, 13a. participant number, 14a. descriptive data, 15. outcome data, 16a. main result, 16b. Category of Continuous variable, 19. limitation, 20. interpretation, 21. generalisation, and 22. funding (items 1 A, 1B, 2, 3, 4, 5, 6, 7, 8, 10, 13a, 14a, 15, 16a, 18, 19, 20, 21, 22). Each item was coded as: Y = present, N = not present, P = partially present, N/A = not applicable, and finally, the percentage of the positive judgement’s total calculation (Table 3). If an article’s total percentage of positive judgement is less than 50%, then it is deemed poor quality and excluded from the study.

**Table 3:** Quality assessment

Strobe Items	1 A	1B	2	3	4	5	6	7	8	10	13 A	14 A	15	16 A	18	19	20	21	22
Andrade et al. (2018)	Y	Y	Y	Y	Y	Y	Y	Y	Y	Y	Y	Y	Y	Y	Y	Y	Y	Y	Y
Edberge et al. (2015)	Y	Y	Y	Y	Y	Y	Y	Y	Y	Y	Y	Y	Y	Y	Y	Y	Y	Y	N
Giuliani et al. (2010)	Y	Y	Y	Y	Y	Y	Y	Y	Y	Y	Y	Y	Y	Y	Y	Y	Y	Y	Y
Horyniak et al. (2016a, 2016b)	Y	Y	Y	Y	Y	Y	Y	Y	Y	Y	Y	Y	Y	Y	Y	Y	Y	Y	Y
Khader et al. (2009)	Y	Y	Y	Y	Y	Y	Y	Y	Y	Y	Y	Y	Y		Y	Y	Y	Y	Y
Massad et al. (2016)	Y	Y	Y	Y	Y	Y	Y	Y	Y	Y	Y	Y	Y	Y	Y	Y	Y	Y	Y
McCann et al. (2016)	Y	Y	Y	Y	Y	Y	Y	Y	Y	Y	Y	Y	Y	Y	Y	Y	Y	Y	Y
McCleary et al. (2016)	Y	Y	Y	Y	Y	Y	Y	Y	Y	Y	Y	Y	Y	Y	Y	Y	Y	Y	Y
Wimann et al. (2017)	Y	Y	Y	Y	Y	Y	Y	Y	Y	Y	Y	Y	Y	Y	Y	Y	Y	Y	N
Wimann et al. (2017)																			
% Positive judgements total /total number of papers	100%	100%	100%	100%	100%	100%	100%	100%	100%	100%	100%	100%	100%	100%	100%	100%	100%	100%	78%

1 A. Title, 1B. Abstract, 2. Background/rationale, 3. Objective, 4. Design, 5. setting, 6. eligibility of the participants, 7. variables, 8. data sources/measurement, 10. study size, 13a. participant number, 14a. descriptive data, 15. Outcome data, 16a. main results, 16b. category of continuous variables, 18. key results, 19. limitations, 20. interpretation, 21. Generalizability, 22. funding. Items, 9, 11, 12 A, 12b, 12d, 13b, 13c, 14b, 14c, 16b, 16c and 17 were not applicable for assessing the papers included in this study. Y present, N not present, P partially present, N/A not applicable, % positive judgements total /total number of papers

Table 3: *Quality Assessment*.

### Data Extraction

Systematic reviews conduct data extraction to minimise human error and bias (Tranfield et al., 2003). The purpose of the data extraction is to directly link to the formulated review question and the planned assessment of the incorporated studies, providing as a visual representation and historical record of decisions made during the process, and as the data-repository for the analysis (Tranfield et al., 2003). Below is the data extraction table developed for this systematic literature review (Table 4). Data extractions contain valuable information such as title, author, findings, concepts, journal, study design, setting, population, and emerging themes.

**Table 4:** Data Extraction

Studies	Title	Objective	Outcome	Prevention Program	Participants’ attitude	Population	Setting	Study design	Concept	Type of study	Journal	Future research
Andrade et al. (2018)	Strategies to Increase Latino Immigrant Youth Engagement in Health Promotion Using social media: Mixed-Methods Study	The objectives of this study were to (1) characterize Adelante participant Facebook reach and engagement and (2) identify post content and features that resulted in greater user engagement.	Post content categories that were statistically significantly associated with post engagement were prevention topics, including substance abuse among others.	Adelante youth intervention and social media	Latino immigrant youth audience in this study had a tendency toward more passive social media consumption	Latino immigrant youth	Washington DC USA	Mixed method	Strategies to increase youth engagement in health promotion	Peer reviewed	JMIR Public Health Surveill	outreach strategies and engagement measurement in future studies.
Edberge et al. (2015)	Defining the ‘community’: Applying ethnographic methods for a Latino immigrant health intervention	Provide brief description of the background for a community level health disparities intervention that aims to help close the gap.	Adelante intervention identified the following disparities: Lack of community attachment, lack of social support and social space, more isolation than connection and racialized identity and exclusion	Adelante youth intervention	Not stated	Latino immigrant youth	Washington DC USA	Ethnographic study	Health disparities	Peer reviewed	Human Organization	N/A
Wimann et al. (2017)	Comorbid psychopathology and everyday functioning in a brief intervention study to reduce khat use among Somalis living in Kenya: Description of baseline multimorbidity, its effects of intervention and its moderation effects on substance use	this paper aimed for assessing baseline multimorbidity and its interactions with a Brief Intervention.	Research found high rates of baseline multimorbidity: 51% (*N* = 168) for depression, 22% (*N* = 74) for PTSD and 23% (*N* = 73) for khat-psychotic symptoms. Group differences. Khat use-time decreased, and functional time increased with significant group interactions (*p* ≤ 0.046). More khat use reduction after the intervention was found (*p* = 0.024).	Brief Intervention	Not stated	Somali refugee youth	Kenya	Random controlled trial	khat use	Peer reviewed	Social Psychiatry and Psychiatric Epidemiology: The International Journal for Research in Social and Genetic Epidemiology and Mental Health Services	Quantitative studies to identify risk and protective factors, in order to inform future interventions. Qualitative studies which identify and include more hidden groups within the community. Inclusion of participants from a range of African countries and distinct tribal/cultural affiliations limits the ability to explore how cultural and religious beliefs.
Giuliani et al. (2010)	Characteristics and prevalence of tobacco use among Somali youth in Minnesota	This paper explores factors related to tobacco use and cessation among Somali youth.	Overall, 22.2% of students reported being ever-users of any form of tobacco. Most notable of these results was the influence of friends and family on lifetime tobacco use. Positive peer pressure and religion appear to be protective factors in tobacco use.	Protective factor: religion and positive peer pressure	Not stated	Somali refugee youth	Minnesota, USA	Mixed method	factors related to tobacco use and cessation.	Peer reviewed	American Journal of Preventive Medicine	
Horyniak et al. (2016a, 2016b)	Heavy alcohol consumption among marginalised African refugee young people in Melbourne, Australia: motivations for drinking, experiences of alcohol-related problems and strategies for managing drinking	This study aimed to describe motivations for drinking, experiences of alcohol-related problems and strategies for managing drinking among marginalized African refugee young people in Melbourne, Australia.	articipants gathered in public spaces to consume alcohol on a daily or near-daily basis. Three key motivations for heavy alcohol consumption were identified: drinking to cope with trauma, drinking to cope with boredom and frustration and drinking as a social experience.	Strategies for managing drinking included attending counselling or residential detoxification programs, self-imposed physical isolation and intentionally committing crime in order to be incarcerated.	Not stated	African refugee youth	Melbourne, Australia	Qualitative study	Substance use	Peer reviewed	Ethnicity & Health	
Khader et al. (2009)	Tobacco use among Palestine refugee students (UNRWA) aged 13–15	The purpose of this paper is to compare tobacco use among Palestine refugee students and students in the general population of the five fields of operation.	In each of the five fields of operation, there was no difference in current cigarette smoking, current use of shisha, or susceptibility to initiate smoking among the three groups of students.	Education, health, relief, and social services	Not stated	Palestinian refugee youth	Gaza Strip, Lebanon, and the West Bank (2005) and in Jordan and Syria (2007)	Two-stage cluster sample Design	Tobacco use	Peer reviewed	Preventive Medicine	
Massad et al. (2016)	Substance use among Palestinian youth in the West Bank, Palestine: a qualitative investigation	This study provides insights into the perceived prevalence and patterns of alcohol and drug use among Palestinian youth.	Most participants reported that substance use exists	Rehabilitation and counselling services	Do not trust the institution, social stigma or do not even know the institution exists.	Palestinian refugee youth	West Bank	Qualitative study	Substance use	Peer reviewed	BMC Public Health	
McCann et al. (2016)	Sub-Saharan African migrant youths’ help-seeking barriers and facilitators for mental health and substance use problems: A qualitative study	Study aimed to identify the help-seeking barriers and facilitators for anxiety, depression and alcohol and drug use problems in young people from recently established sub-Saharan African migrant communities.	Four help-seeking barriers were identified: stigma of mental illness, lack of mental health literacy in parents and young people, lack of cultural competency of formal help sources, and financial costs deterring access. Five help-seeking facilitators were abstracted: being open with friends and family, strong community support systems, trustworthiness and confidentiality of help-sources, perceived expertise of formal help-sources, increasing young people’s and parents’ mental health literacy.	Educating people about the important mental health treatment	Do not seek help	Sub-Sahara African refugee youth	Melbourne, Australia	Qualitative study	Barriers and facilitators for mental health and substance use problems	Peer reviewed	BMC Psychiatry	
McCleary et al. (2016)	Connecting Refugees to Substance Use Treatment: A Qualitative Study	Study explores factors that support and prevent refugees from connecting with chemical health treatment.	Lack of culturally informed treatment models, policy issues, and client characteristics act as barriers to engaging with treatment. Ongoing case management and coordination were identified as important to successful linkage.	Substance use intervention	Some participants are not motivated to seek treatment.	Refugee youth	Midwest, USA	Qualitative study	Substance use treatment	Peer reviewed	Social Work in Public Health	

### Study Characteristics

#### Study Objectives and Designs

The study designs include four qualitative, one ethnographic, two mixed methods, one random controlled trial, and one two-cluster sample. The studies were published in nine different journals (Table 5).

**Table 5:** Summary of the findings

No.	Studies	Outcome	Prevention Program	Participant’s attitudes
1	Andrade et al. (2018)	Post content categories that were statistically significantly associated with post engagement were prevention topics, including substance abuse among others.	Adelante youth intervention and social media	Latino immigrant or refugee youth audience in this study tended toward more passive social media consumption
2	Edberge et al. (2015)	Adelante intervention identified the following disparities: Lack of community attachment, lack of social support and social space, more isolation than connection and racialized identity and exclusion	Adelante youth intervention	Not stated
3	Giuliani et al. (2010)	Overall, 22.2% of students reported being ever-users of any form of tobacco. The most notable of these results was the influence of friends and family on lifetime tobacco use. Positive peer pressure and religion appear to be protective factors in tobacco use.	Protective factor: religion and positive peer pressure	Not stated
4	Horyniak et al. (2016a, 2016b)	participants gathered in public spaces to consume alcohol on a daily or near-daily basis. Three key motivations for heavy alcohol consumption were identified: drinking to cope with trauma, drinking to cope with boredom and frustration and drinking as a social experience.	Strategies for managing drinking included attending counselling or residential detoxification programmes, self-imposed physical isolation and intentionally committing the crime to be incarcerated.	Not stated
5	Khader et al. (2009)	In each of the five fields of operation, there was no difference in current cigarette smoking, current use of shisha, or susceptibility to initiating smoking among the three groups of students.	Education, health, relief, and social services	Not stated
6	Massad et al. (2016)	Most participants reported that substance use exists	Rehabilitation and counselling services	Do not trust the institution, social stigma or do not even know the institution exists.
7	McCann et al. (2016)	Four help-seeking barriers were identified: stigma of mental illness, lack of mental health literacy in parents and young people, lack of cultural competency of formal help sources, and financial costs deterring access. Five help-seeking facilitators were abstracted: being open with friends and family, strong community support systems, trustworthiness and confidentiality of help-sources, perceived expertise of formal help-sources, increasing young people’s and parents’ mental health literacy.	Educating people about the important mental health treatment	Do not seek help
8	McCleary et al. (2016)	Lack of culturally informed treatment models, policy issues, and client characteristics act as barriers to engaging with treatment. Ongoing case management and coordination were identified as important to successful linkage.	Substance use treatment	Some participants are not motivated to seek treatment.
9	Wimann et al. (2017)	Research found high rates of baseline multimorbidity: 51% (*N* = 168) for depression, 22% (*N* = 74) for PTSD and 23% (*N* = 73) for khat-psychotic symptoms. Group differences. Khat use-time decreased, and functional time increased with significant group interactions (*p* ≤ 0.046). More khat use reduction after the intervention was found (*p* = 0.024).	Brief Intervention	Not stated

#### Study Setting and Participants

Nine peer-reviewed articles met the inclusion criteria for this systematic literature review. They were published from 2009 to 2020. Four studies were conducted in the USA, two in Australia, two in the Middle East, and one in Kenya. Participants in these studies are refugees youth from these host countries.

### Findings

#### Substance Use Prevention Programs

The findings revealed a gap in the literature about substance use prevention programs amongst refugee youth. In the nine articles that met the inclusion criteria for this study, only two substance use prevention programs emerged. The substance use prevention programs identified in the study included Adelante Social and Marketing Campaign (ASMC) and Screening and Brief Intervention (SBI).

ASMC is a community-based intervention program offered by the Advance Centre for the Advancement of Immigrant/Refugee Health in Washington, DC, USA. This is a well-known primary prevention program, which addresses risk factors for substance use and other co-occurrences amongst Latino adolescents aged 12 to 19 years in a suburb of Washington, DC (Andrade et al., 2018; Edberg et al., 2015). The study employed the 4-year Adelante primary prevention program to address risk factors for substance use and other issues amongst Latino adolescents, aged 12 to 19 years (Andrade et al., 2018). In the two studies, ASMC was used to investigate two distinct scenarios. Firstly, it was used to identify post contents and features that resulted in greater user engagement (Andrade et al., 2018). Secondly, Edberg et al. (2015) used ASMC to provide a brief description of the background for community-level health disparities intervention that aims to help close the gap. The intervention is organised in a group of one to five short psychotherapeutic sessions for substance users (Karno et al., 2021; Widmann et al., 2017). Participants engage in a standardized screening for substance use problems, receive systematic feedback on substance-related risks, and participate in a motivational intervention to reduce substance use (Saitz, 2014).

On the other hand, SBI is used by non-psychiatric healthcare providers for substance use prevention. The approach relies on motivational interviewing focusing on empowering patients during the intervention (Karno et al., 2021; Widmann et al., 2017). SBI was successfully used to assist refugee youth in addressing substance use issues.

Six studies explore the strategies and protective factors for substance use prevention. Giuliani et al. (2010) and McCann et al. (2016) identified protective factors that influence the cessation of substance use amongst refugee youth, including strong community support systems, family, and friends. Protective factors such as trustworthiness, confidentiality of help sources, perceived expertise of formal help sources, and increasing young people’s and parents’ substance use literacy play a vital role in reducing the initiation and maintenance of substance use. Research has shown that providing refugee youth woth counselling, ongoing case management coordination, residential detoxification programmes, and individual strategies such as self-imposed physical isolation can mitigate substance use amongst them (Horyniak et al., 2016a; McCleary et al., 2016). Moreover, researchers identified protective factors including academic success, and participation in voluntary activities can assist in reducing substance use (Massad et al., 2016).

The findings highlight protective factors that shield refugee youth from substance-use. These protective factors included religion, positive peer pressure, health, relief, and social services (Giuliani et al., 2010; Khader et al., 2009; McCann et al., 2016). More importantly, connecting with substance use treatment is suggested to be one way refugee youth can reduce substance use (McCann et al., 2016; McCleary et al., 2016).

#### Participants’ Attitudes toward Substance use Prevention Programs

The studies that attempt to investigate the attitude of refugee youth towards substance use prevention programs have revealed mixed results. First and foremost, refugee youth demonstrated a lack of confidence in the institution that provides substance use prevention programs (Massad et al., 2016; McCann et al., 2016). For instance, refugee youth in substance use treatment expressed a sense of scepticism towards the institution that provides counselling and rehabilitation (McCann et al., 2016; McCleary et al., 2016). Other researchers found out that refugee youth’s participation in substance use treatments is not motivated and therefore they are too reluctant to seek treatment (McCann et al., 2016; McCleary et al., 2016). While other research shows that refugee youth are unaware of any local institutions to support youth with substance use problems (Massad et al., 2016). The refugee youth who participated in the Adelante intervention and utilise social media demonstrated a positive propensity towards engaging in more passive forms of social media usage (Andrade et al., 2018).

#### Outcomes of Substance Use Prevention Programs

ASMC showed that prevention topics were significantly associated with post-engagement behaviour, such as substance use (Andrade et al., 2018). ASMC also identified the inequalities that promote substance use amongst the refugee youth such as a lack of community attachment, social support and social space, isolation rather than connection, and a racialized identity (Andrade et al., 2018; Edberg et al., 2015). The study indicated lack of social space leading to refugee youth finding sanctuary in gang activities (Edberg et al., 2015). ASMC also indicated that the most engaging topic discussed in social media posts was substance use prevention, which accounted for 8.4% of the posts with the p-value < 0.001 (Andrade et al., 2018).

The outcome for SBI was significant. The findings indicate that there was a decline in the amount of time that refugee youth spent using substances as their functional time increased among refugee youth (Widmann et al., 2017). As a result, SBIs appear to reduce substance use to some extent.

## Discussion

### Overview of the Findings

The study aimed to explore different substance use prevention programs, summarise refugee youth’s attitudes towards these programs and outline the outcomes of the prevention programs. This systematic literature review appeared to be the first of its kind to systematically synthesis substance use prevention programs amongst refugee youth. The findings from this study supported the hypothesis that research on substance use prevention programs amongst refugee youth is scarce. Only two substance use prevention programs were identified in the study: SBI and ASMC. Although ASMC was included in only one study on substance use prevention programs, its main objectives were to identify the activities in which refugee youth participate and to outline potential areas for intervention. ASMC did not employ strategies to reduce substance use. Moreover, most studies included in this context outlined strategies and protective factors that assist in reducing substance use and related consequences amongst refugee youth. If refugee youth adhere to protective factors such as family attachment, religion, and commitment to social norms, then there is a likelihood that they can avoid the initiation and maintenance factors of substance use. Another important strategy that emerges from this study is the need to increase refugee youth and parents’ substance use literacy. Increasing literacy can help refugee youth to understand the risk substance use can have on their health, social interactions, and economic wellbeing.

Previous studies asserted that the efficacy of substance use prevention program depends on the participants’ attitude towards intervention and its outcomes (Espada et al., 2015). However, what is alarming is refugee youth have a negative attitude about institutions providing substance reduction services. Although the ASMC and SBI demonstrated positive outcome, such an approach can be associated with high dropout rates and subsequently, poor outcomes in substance use prevention programs. Individuals who have confidence in professional services are more likely to seek assistance and therefore, reduce substance use.

### Implication

The dearth of research on substance use prevention programs programmes may have significant ramifications, considering the substantial body of literature indicating the widespread occurrence of substance use amongst refugee youth. There exists convincing evidence that the refugee youth cohort could be at risk of substance use disorders but are not seeking help. Substance use has a debilitating impact on an individual’s health, social and economic well-being. For refugee youth not seeking assistance to reduce substance use may indicate they are suffering significant consequences on top of their challenges before and after migration.

Previous studies conducted on youth in general has identified many substance use prevention programs in the literature that can mitigate the prevalence of substance use and related consequences (Barrett et al., 1988). However, little is known in the literature about the extent and effectiveness of substance use prevention programs including individual and group counselling, alternative programs, and family and community interventions, applicable for refugee youth (Barrett et al., 1988; Foss-Kelly et al., 2021; Radoi, 2014). Researchers only indicated that refugees are aware of some substance use treatment services. There are substantial differences between being aware of a service and actively interacting and engaging with it. Therefore, it is significant for refugee youth to be aware of substance use prevention programs and seek assistance to reduce substance dependence.

Refugee youth’s lack understanding of substance use prevention programs might be compounded by their inability to seek professional help. Scholarly literature suggest that refugee youth do not seek professional help because of barriers including lack of understanding of the new health system, poor mental literacy, language problem, limited transportation and cultural differences (Posselt et al., 2014; Shaw et al., 2019). Additionally, young refugees, particularly those who are forced to flee their countries due to persecution or violence, frequently encounter substantial trauma and stress without adequate access to mental health services. The pressures encompass a dearth of livelihood opportunities, familial separation, risky journeys, and vulnerability to assault and abuse. Despite managing to escape life-threatening situations in their native countries, these youth individuals often face further prejudice and become targets of in their host countries. They frequently encounter challenges accessing appropriate services, especially when it comes to disparities in mental healthcare services caused by socio-cultural factors. While additional resources and support are necessary, it is crucial to provide culturally sensitive and customised interventions to refugee youth.

## Conclusion and Future Research

In conclusion, prevention programs for substance use remain obscure despite the prevalence of substance use amongst refugee youth. The prominent finding of this review is that the majority of the investigations failed to address substance use prevention programs, as their focus was primarily on protective factors and strategies to reduce substance-use. While the study does make an attempt to address substance use prevention programs, it also incorporates other risk behaviours as well. In such investigations, it is difficult to deduce the outcome and attitudes of the participants. Future research is warranted regarding the implementation of substance use prevention programs amongst refugee youth. The findings are an indication of the need to conduct a robust substance use prevention program such as individual and group counselling, alternative programs, and family and community interventions tailored specifically to refugee youth. Furthermore, research should demonstrate the efficacy of each substance use prevention program by exploring participants’ attitudes towards intervention and measuring the outcome of the study. This can fill the gap in the literature with empirical evidence on how refugee youth participate in substance use prevention programs and maximise the benefits by reducing substance use.

## Limitation

It is essential to acknowledge the limitations of this study. The primary constraint lies in the study’s narrow focus on refugee youth, restricting the search to this specific keyword. Consequently, fewer articles satisfied the inclusion criteria. The study may have overlooked relevant articles that employ alternative terms such as ‘immigrant’, ‘migrant’, or ‘cultural linguistic diverse individuals’. Using broader and more inclusive terms can improve the quality of future research by redesigning the search strategy. .
